# Does breast cancer policy meet the needs of Aboriginal and Torres Strait Islander women in Australia? a review

**DOI:** 10.1186/s12939-023-01941-3

**Published:** 2023-07-05

**Authors:** Vita Christie, Lynette Riley, Deb Green, Kylie Snook, Mandy Henningham, Boe Rambaldini, Janaki Amin, Chris Pyke, Megan Varlow, Sally Goss, John Skinner, Ross O’Shea, Deb McCowen, Kylie Gwynne

**Affiliations:** 1 Djurali Centre for Aboriginal and Torres Strait Islander Research and Education, Macquarie University, 6 First Walk, Sydney, NSW 2109 Australia; 2 Indigenous Studies & Aboriginal Education, The University of Sydney, Education Building (A36), Sydney, NSW 2006 Australia; 3 Armajun Aboriginal Health Service, Rusden Street, Armidale, NSW 2350 Australia; 4 BreastSurgANZ, Black Rock, Melbourne, Victoria 3193 Australia; 5 Cancer Council Australia, Level 2, 320 Pitt Street, Sydney, NSW 2000 Australia; 6 Department of Health Sciences, Macquarie University, 75 Talavera Road, Sydney, NSW 2109 Australia; 7 Mater Hospital, Vulture Street, South Brisbane, QLD 4101 Australia; 8 Cancer Council Australia, Level 2, 320 Pitt Street, Sydney, NSW 2109 Australia; 9 University of Sydney, Sydney, NSW 2006 Australia; 10 Foundation of Breast Cancer Care and Breast SurgANZ, Black Rock, Melbourne, Victoria 3193 Australia; 11 Armajun Aboriginal Health Service, Rivers St, Inverell, NSW 2360 Australia

**Keywords:** Aboriginal and Torres Strait Islander, Indigenous, Health, Policy, Breast cancer

## Abstract

**Objective:**

To evaluate if existing Australian public policy related to screening, diagnosis, treatment and follow up care for breast cancer addresses the needs of and outcomes for Indigenous^1^ women?

**Methods:**

This review of policy employed a modified Delphi method via an online panel of experts (n = 13), who were purposively recruited according to experience and expertise. A series of online meetings and online surveys were used for data collection. The aims of the study were to: Identify all existing and current breast cancer policy in Australia;  Analyse the extent to which consideration of Indigenous peoples is included in the development, design and implementation of the policy; and Identify policy gaps and make recommendations as to how they could be addressed. The policies were evaluated using ‘A Guide to Evaluation under the Indigenous Evaluation Strategy, 2020’.

**Results:**

A list of current breast cancer policies (n = 7) was agreed and analysed. Five draft recommendations to improve breast cancer outcomes for Indigenous women were developed and refined by the panel.

**Conclusions:**

Current breast cancer policy in Australia does not address the needs of Indigenous women and requires change to improve outcomes.

## Introduction

### Breast cancer in Indigenous women in Australia

Breast cancer is the most diagnosed cancer affecting Australian women, and the second largest cause of cancer death in Australian women. While the incidence rate of breast cancer is lower in Indigenous women that non-Indigenous women [1], the mortality rate is higher, with Indigenous women 1.2 times more likely to die from the disease [2]. In New South Wales, Indigenous women are 69% more likely to die from their breast cancer than non-Indigenous women [3]. There are a number of factors that may contribute to this higher mortality rate, including lower participation in screening services, socioeconomic disadvantage, younger age at diagnosis, geographic remoteness, co-morbidities and a more advanced stage of cancer at the time of diagnosis [1, 3, 4]. It is not thought to be due to differences in histological subtype or mammographic density [5, 6].

### The role of health policy in Australia

The Australian Policy handbook defines policy as “A statement of government intent, and its implementation through the use of policy instruments.”[7]. In Australia, government policy at the macro level is presented as legislation. Funding typically flows directly from legislation. In order for funding to be allocated, there needs to be a program and associated legislation and policy. Policy is critical to funding, priorities and outcomes, and representation of the issues facing Indigenous women in policy is vital to those issues being addressed in service delivery models and approaches.

### Research question

In order to refine our research question, we used PICO format:

- Patient/population: Aboriginal and Torres Strait Islander women.
- Intervention: review Australian breast cancer policy to assess whether it meets the need of the patient/population.
- Comparison: non-Indigenous women in Australia.
- Outcomes: Expert consensus on recommendations for improvement of breast cancer policy in Australia.

Research Question: How does existing Australian policy related to screening, diagnosis, treatment and follow up care for breast cancer address the needs of and outcomes for Indigenous women?

Hypothesis for the study: that existing/current policy for breast cancer in Australia does not adequately address the needs of Indigenous women. And that identifying policy gaps can inform future implementation and improve outcomes for Indigenous women In Australia.

### Aims

- Identify all existing and current policy.
- Analyse the extent to which consideration of Indigenous peoples is included in the development and design of the policy.
- Identify policy gaps and make recommendations as to how they could be addressed.

### The Delphi Method

The Delphi method- commonly used in reviews- relies on consensus among experts (often referred to as a panel) to answer a research question [8]. Despite variation in design, the Delphi method is based on a series of questions put to the expert panel, whereby the panel members are surveyed for opinions on a particular issue. The questions follow a sequential process, whereby each round/survey is partially based findings of the previous round/survey, allowing for evolution of the study [8]. Results of each round are transparent and deidentified, encouraging reflection on the views of others and possible readjustment of their own perspectives [9]. The deidentification of responses provide protection from bias or perceived negativity [8]. The Delphi method is a framework - where each round augments the previous round - is designed to result in a consensus view.

We have used the Delphi method to look at breast cancer policy; identifying the gaps in policy and synthesizing the evidence regarding the needs of Indigenous women to provide recommendations for policy improvement.

## Methodology

### Delphi Method

**Fig. 1:**
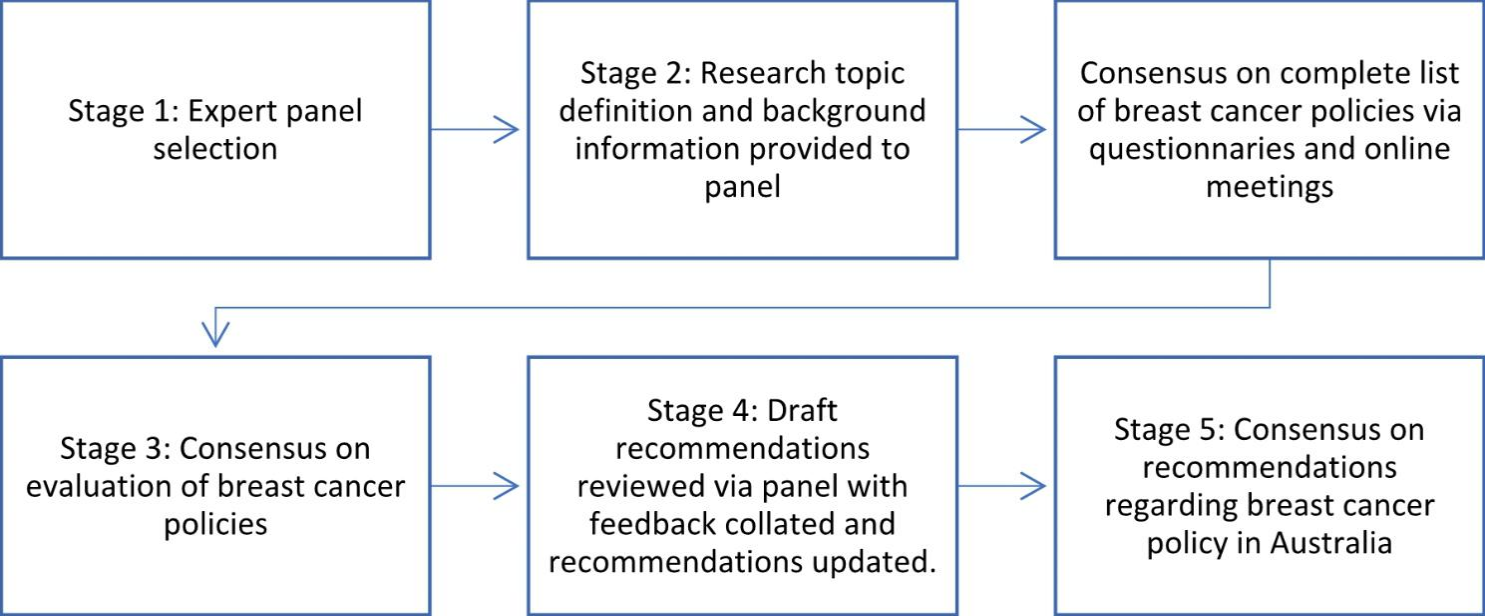
Delphi process

The study utilised the Delphi method via an online panel [10] (see Fig. 1). The panel were purposively recruited by inviting people with knowledge and experience in breast cancer policy and practice, specifically representatives of the breast cancer peak organisations, representatives of the Aboriginal Community Controlled Health sector, consumers and experts in public health policy. The Delphi method was undertaken in five stages over a three month period.

**Fig. 2:**
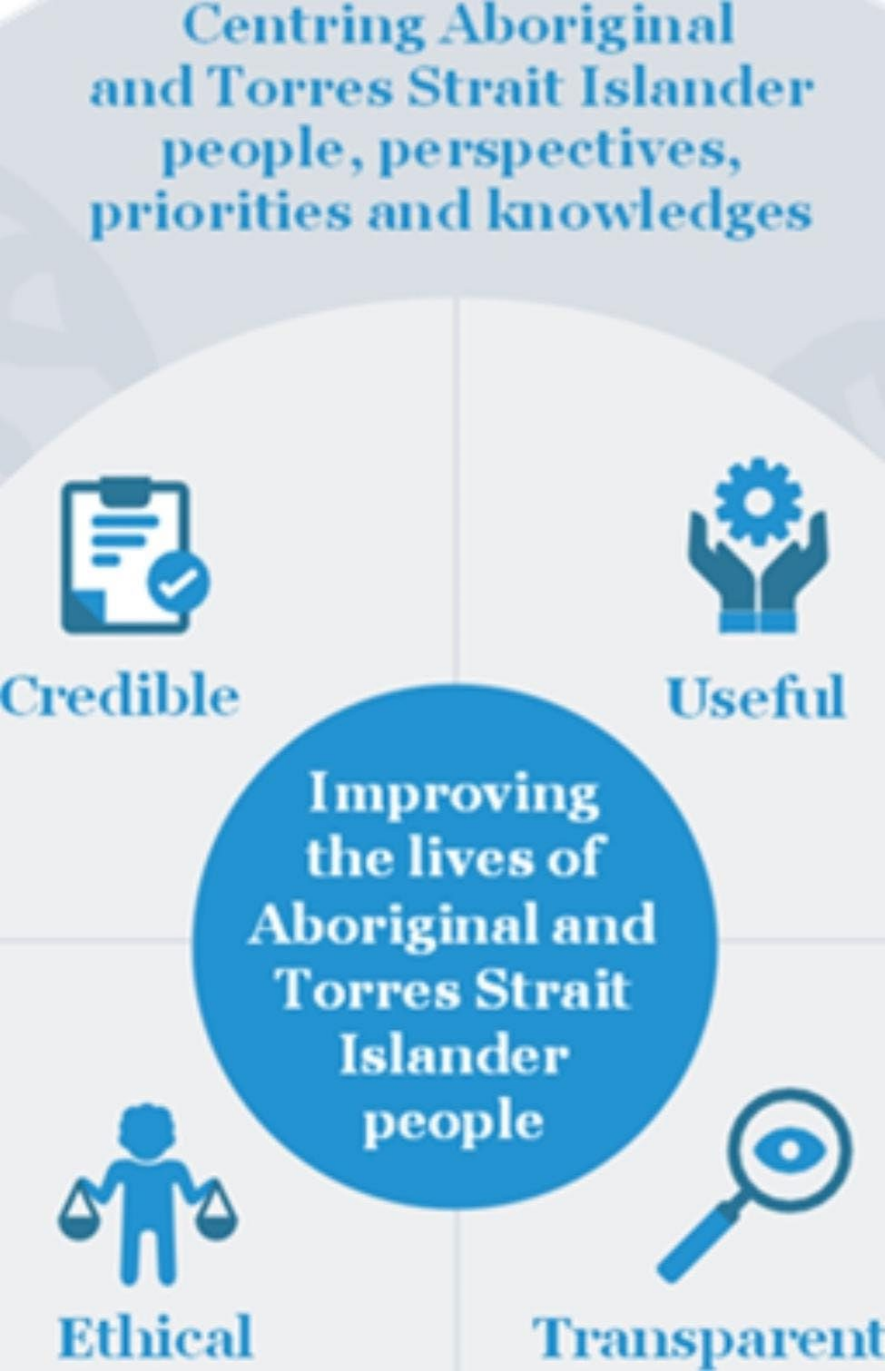
Guiding principles for the Indigenous Evaluation Strategy

### Search strategy and selection criteria

A systematic hand search of the grey literature (utilising Google search engine) was conducted using the following search terms: “Breast cancer Australia policy”; “Australia breast cancer standard policy”; “Australia breast cancer funding policy”. Table 1 shows inclusion and exclusion criteria were used to select the policies for the study.

**Table 1:** Inclusion and exclusion criteria

Inclusion criteria	Exclusion criteria
• Breast cancer • Screening, diagnosis, treatment, follow up • Policy • Australia only • Current (not been replaced/updated) • Publicly available • Document includes consequences for relevant organisations for non-compliance	• Cancer in general; other cancers • Guidelines; standards; guidance; strategy; position statement; fact sheets • Information only without specific policy; • International policy • Accreditation standards • Outdated/replaced policies

### Stage 1

In the first stage or our review, one author (VC) conducted an extensive search (as detailed above) of the grey literature to assemble a list of current policies for breast cancer in Australia. This search was conducted over multiple days during the month of September 2022, employing the stopping rule of ten pages searched (and no mention of policy on pages). The definition of ‘current’ is: an active policy, which has not been superseded. The policies were collated into a spreadsheet with: owner; source; scope; purpose; commencement date; and whether the policy was current or not, extracted.

Invitations to ‘experts’ (see definition above) were sent out via letters/email giving context to the study and a deadline for acceptance. The organisations approached were as follows:

Breast Cancer Network Foundation, Cancer Australia, Cancer Council, Foundation for Breast Cancer Care, Breast SurgANZ, Cancer Institute and an Aboriginal Community Controlled Health Organisation.

### Stage 2

The second stage of the review comprised two online meetings with members of the expert panel. These meetings served as an introduction to the project and a review of the collated breast cancer policies, to ascertain if the list was complete. The definition of ‘policy’ (see Results) was refined over several meetings.

### Stage 3

The third stage was a process of evaluation to examine how well the policies addressed the needs of Indigenous women. Two authors (VC, KG) of the paper evaluated each policy according to the Australian Government Productivity Commission’s: A Guide to Evaluation under the Indigenous Evaluation Strategy, 2020 [11], specifically Fig. 2 [11].

This evaluation was added to the spreadsheet of policies. Under the following broad categories of: Credible/Useful/Ethical/Transparent, each category was scored either: Yes/No/Partially. Definitions of the categories were as followed:

Credible: trustworthy; grounded on rigorous methodology, and includes Indigenous values, perspectives and knowledges.

Useful: considers the needs of a range of end-users (primarily that of Indigenous people) and of high quality, with findings are available to decision makers, and timed to contribute to policy-making and implementation decisions.

Ethical: improves the quality and consistency of evaluation and ensures that it has a positive impact on Indigenous people.

Transparent: open and honest in intention and information, specifically providing access for Indigenous people.

The evaluation spreadsheet was then disseminated amongst the expert group along with an online survey and each member was invited to provide feedback via the survey and/or an online meeting. The survey and follow up meeting provided further review and discussion about the policies and scoring with a view to reaching consensus.

### Stage 4

Stage four consisted of the development of draft recommendations to improve breast cancer policy in Australia. On close examination of the complete list of breast cancer policies and comparing with the evidence of what will improve outcomes for Indigenous women with breast cancer [12], one author (VC) drafted five recommendations in response to the policies and their perceived shortcomings.

The draft recommendations were presented to the panel via an online meeting and/or an online survey for feedback. The feedback was then collated with further discussion and input from the panel to reach consensus where possible. Where consensus was not possible, it was reported.

### Stage 5

In the fifth and final stage of the policy review, the first author sent the draft recommendations survey results and comments to the panel, with responses to each comment. Feedback from this process was then incorporated into the draft review of breast cancer policy in Australia and, following discussion of the panel, consensus was reached on newly worded recommendations.

## Results

### Stage 1

Initially, one author (VC) invited 21 people to be part of the expert group, with a range of expertise and experience (summarised in Table 2. Deidentified experience of panel members), and 14 accepted and participated. One member withdrew from the panel midway through the process due to time constraints, with a total of 13 panel members remaining (not including VC and KG).

**Table 2:** Deidentified experience of panel members

Panel member	Area/s of expertise
1	Aboriginal Community Controlled Health Service sector; Public policy
2	Breast cancer specialist; Breast Cancer peak organisation representative
3	Breast Cancer peak organisation representative; Public policy
4	Breast Cancer peak organisation representative; Public policy
5	Breast cancer specialist; Academic
6	Public policy; Academic; Consumer
7	Breast cancer specialist; Breast cancer peak organisation representative
8	Breast cancer peak organisation representative; Public policy
9	Public Policy; Aboriginal Community Controlled Health Service sector; Academic
10	Aboriginal Community Controlled Health Service sector; Public policy; Academic
11	Aboriginal Community Controlled Health Service Sector; Consumer
12	Breast Cancer peak organisation representative; Academic
13	Aboriginal Community Controlled Health Service Sector; Academic

### Stage 2

The agreed definition of policy: “A publicly available statement of intent or expected practice that is not negotiable and requires mandatory compliance (with consequences if not complied with).”

The agreed list of breast cancer policies is shown in Table 3, with evaluation columns added (from Stage Three). There was 100% agreement on the list of policies from the panel.

**Table 3:** Final list of breast cancer policies (Stage 1), evaluated (Stage 3)

Name of policy	Owner	link	Scope	Purpose	Commencement date	Credible	Useful	Transparent	Ethical
National Cancer Control policy- breast cancer	Cancer Council	https://www.cancer.org.au/about-us/policy-and-advocacy/early-detection-policy/breast-cancer-screening	general public	Presents information about breast cancer screening and related policy	2014	no	no	yes	no
Breast Cancer screening policy	Cancer Council	https://www.cancer.org.au/about-us/policy-and-advocacy/early-detection-policy/breast-cancer-screening	general public	Encourage early detection	2018	no	no	no	no
BreastScreen Australia National Eligibility Policy	BreastScreen/Department of Health, Australia	https://www.health.gov.au/resources/publications/breastscreen-australia-national-eligibility-policy	general public	To establish who is eligible for free screening	2018	no	partially	no	no
BreastScreen Australia – Policy on screening women aged 40–49 years	BreastScreen/Department of Health, Australia	https://www.health.gov.au/resources/publications/breastscreen-australia-policy-on-screening-women-aged-40-49-years	general public	Evaluation of the BreastScreen Australia program’s expansion of the target age for active recruitment to include women aged 70–74 years	2018	no	no	no	no
BreastScreen Australia – Policy on screening versus diagnostic mammography	BreastScreen/Department of Health, Australia	https://www.health.gov.au/resources/publications/breastscreen-australia-policy-on-screening-versus-diagnostic-mammography	general public	Policy on screening versus diagnostic mammography	2013	no	no	no	no
BreastScreen Australia – Policy and practice in relation to symptomatic women	BreastScreen/Department of Health, Australia	https://www.health.gov.au/resources/publications/breastscreen-australia-policy-and-practice-in-relation-to-symptomatic-women	general public	Policy and practice in relation to symptomatic women in BreastScreen Australia	2019	no	no	no	no
Policy-at-a-glance – Breast Cancer Screening (Mammography) Policy	Public Health Assocation Australia	https://www.phaa.net.au/documents/item/1666	Federal, State and Territory Governments, policy makers, cancer organisations and screening program managers.	Outlines the evidence and makes suggestions for future research and the development of best-practice guidelines.	2016	no	no	no	no

### Stage 3

Using the broad categories of the Australian Government Productivity Commission’s: A Guide to Evaluation under the Indigenous Evaluation Strategy, 2020, only one of the policies was deemed Transparent and only one of the other policies was deemed Partially Useful. None of the policies were deemed Ethical or Credible. The panel agreed 100% with the assessment of the policies.

### Stage 4

The following five draft recommendations were sent to the panel as an online survey with a brief explanation/justification and there were three options for answers: Agree *or* Disagree and Comments. In Table 4, we have recorded the number of respondents who agreed or disagreed to each of the recommendations.

**Table 4:** First round of draft recommendations

Draft recommendation	Brief explanation/justification	% agreement by panel responses	Number of respondents to Agree/Disagree to the recommendation	
1.That Aboriginal and Torres Strait Islander people are consulted in formation of policy	Much of the policy has no mention of Aboriginal and Torres Strait Islander women and the difference in breast cancer outcomes so does not meet the needs of Aboriginal and Torres Strait Islander women.	100%	10	
2.Screening free and accessible at earlier age	Aboriginal and Torres Strait Islander women more likely to suffer at younger ages than non-Aboriginal and Torres Strait Islander women.	87.5%	7	
3.That women with symptoms are not discouraged from attending Screening	Current policy is that women with symptoms do not attend screening; however for a group that has under-representation in screening, it is vital that they are welcomed to screening or given a clear alternate pathway via policy.	100%	7	
4.It is not enough to acknowledge the barriers for Aboriginal and Torres Strait Islander women and not address them or provide appropriate solution	Several polices refer to the specific barriers faced by Aboriginal and Torres Strait Islander women, however, there are no official policies to deal with these barriers.	100%	8	
5.Policy should not be based on assumption of adherence to guidelines by the consumer	One of the policies states : “To be effective on a population basis, a high compliance rate of attendance of women in the appropriate age range for screening mammography is necessary”.	85.71%	7	

Ten (of the 14) members of the panel responded to the survey, either agreeing, disagreeing or neither agreeing nor disagreeing (leaving a comment instead).

### Stage 5

The first author collated the panel’s comments regarding the draft recommendations. The first author then responded to these comments and added a Result column to mark whether or not consensus was reached and wording of the recommendation remained the same, or consensus was not reached and extra work needed, as shown in Table 5.

**Table 5:** Survey results of draft recommendations, with responses

Draft recommendation (with explanation/justification)	*Comment/s by the expert panel*	Response to comments	Result
1. That Aboriginal and Torres Strait Islander women are consulted in the formation of policy (Much of the policy has no mention of the difference in outcomes for Aboriginal women and therefore does not address needs of Aboriginal and Torres Strait Islander women)	*No comments*		Consensus reached.
2. Screening for breast cancer is free and accessible at an earlier age. (Aboriginal and Torres Strait Islander women are more likely to be diagnosed at a younger age than non-Aboriginal and Torres Strait Islander women)	*Does earlier screening result in better outcomes or result in increased adverse effects of screening: detection of indolent cancers; increase burdens associated with diagnosis. Have they been appropriately assessed with Aboriginal and Torres Strait Islander women?*	According to evidence that has been gathered relevant to early detection of breast cancer for Aboriginal and Torres Strait Islander women, there has only been one paper found in the grey literature that reports on proportion of recalled cases, leading to the assumption this has not been extensively examined. There is evidence, however, that Aboriginal women are more likely to be younger at diagnosis.	Further research needed about the age to commence screening for Indigenous women although it was agreed that earlier screening was likely to be beneficial.
3. That women with symptoms are not discouraged from attending Screening (Current policy is that women with symptoms do not attend screening; however, for a group that has under-representation in screening, it is vital that they are welcomed to screening or given a clear alternate pathway via policy)	*There are very good reasons for this: no diagnostic workup equipment/no doctors present. However examination of how this issue can be handled in remote areas should be closely examined*	Acknowledging that there are good reasons to dissuade those with symptoms from attending screening, we still believe that the message that this policy conveys is problematic from the point of view of those that experience more barriers than the majority of women in Australia. That the policy simply states what not to do, without explicitly stating the other options means it acts as a deterrent.	Agreed that Indigenous women should not be dissuaded from attending screening and should have a clear pathway to care should they have symptoms. Consensus not reached on the recommendation itself.
	*I agree with the second part of the statement - that women with symptoms are welcomed and given an alternative pathway via policy. But don’t agree they should screen anyway as the screening program is designed for asymptomatic women and women with symptoms need a different approach.*		
	*The current National screening programme is geared towards women of low risk. I think Aboriginal and Torres strait Islander people should have an alternate pathway. When examining the different state’s Breastscreen organisations there are better systems for implementation to help with compliance specifically for Aboriginal and Torres Strait islander women (look at Victoria and NSW). Maybe these state by state approaches need to be harnessed for a standardised nation approach for this at-risk population ?????*		
4. It is not enough to acknowledge the barriers for Aboriginal and Torres Strait Islander women and not address them or provide appropriate solutions (Several polices refer to the specific barriers faced by Aboriginal and Torres Strait Islander women, however, there are no official policies to deal with these barriers)	*Unsure how a policy addresses the barriers but happy to learn. generally it requires great flexibility and often creativity to adapt guidelines to suit individual circumstances*	The concern is that if policy does not address barriers, then health practitioners are not provided solutions, nor are they beholden to them. Adapting guidelines is fine but as the name suggests, guidelines are to guide, they are not a mandate. As defined in our Review process, a policy is: A publicly available statement of intent or expected practice that is not negotiable and requires mandatory compliance (with consequences if not complied with). Our definition of guidelines would be significantly different. If we have an alternate pathway for Aboriginal and Torres Strait Islander people, then it should be made into a policy, not simply a system of implementation.	Agreed that the national policy level can learn from state-based guidelines. Consensus reached that policy must provide solutions.
	*The current National screening programme is geared towards women of low risk. I think Aboriginal and Torres strait Islander people should have an alternate pathway. When examining the different state’s Breastscreen organisations there are better systems for implementation to help with compliance specifically for Aboriginal and Torres Strait islander women (look at Victoria and NSW). Maybe these state by state approaches need to be harnessed for a standardised nation approach for this at-risk population ?*	We agree that the national screening program needs to be adapted to better suit the needs of all women, not just low risk women. It is also true that there are variations in state approaches, however policy takes place at the national level and therefore this is where the change needs to be mandated. If we could learn from states that are better adapted at the guideline level and translate it into national policy, this would be ideal.	
5. Policy should not be based on assumption of adherence to guidelines by the consumer (One of the policies states: “To be effective on a population basis, a high compliance rate of attendance of women in the appropriate age range for screening mammography is necessary”)	*This is simply a global public health policy statement. Having worked in the public health industry I do not read this as meaning the women will come forward without multiple levels of health promotional strategies*	We believe it is not enough for policy to be based on resources brought forward by the consumer. This is not realistic and particularly so for those who are underrepresented in the system. While it might be a global public health statement, it has been incorporated into a policy, thereby transferring responsibility to the consumer.	Policy must inform health practice, not depend on the responsibility of the consumer.
	*The statement, and explanation and both dependent on a clear definition of ‘effectiveness’.*	The definition of ‘effectiveness’ as stated by the policy: “Where screening mammography has been provided in an organised and systematic manner it has been shown to be effective in decreasing mortality from breast cancer by around one third to a half in women over 50 years of age who regularly attend.” Again, this definition is reliant on the consumer bearing responsibility.	
	*Agree, and would like to see policy put in place to support increased participation across the system rather than making it an individual responsibility or issue of ‘compliance’. Most women when asked would like to screen, it is just that other things are a higher priority or create barriers to screening*	Agree	
	*This talks to public funding of a screening program- screening for aboriginal women should not have the same KPIs as non aboriginal women when it comes to funding*	The aim of this review is to incorporate the needs of Aboriginal and Torres Strait Islander into nation-wide policy. We believe that developing separate KPIs risks not capturing the needs of other minority groups, and also has the potential to lower expectations when it comes to Aboriginal and Torres Strait Islander women and ‘others’ the women. It’s possible to do but hard to achieve unless there is a conscious decision/effort.	
	*Particularly for this group*	Agree	

As a result of the first part of Stage Five there was one agreed recommendation and four partially agreed recommendations, with further discussion to reach consensus on the final list of recommendations.

### Agreed recommendation

1. *That Aboriginal and Torres Strait Islander women are consulted in the formation of policy.*

### Partially agreed recommendations with outcomes

1. *Screening for breast cancer is free and accessible at an earlier age.*

As mentioned in Table 5, there is scant evidence relating to the early detection of breast cancer for Aboriginal and Torres Strait IslanderI women [13]. There was mention of only one paper found in the grey literature reporting on proportion of recalled cases, leading to the assumption this has not been extensively examined [14]. There is evidence, however, that Indigenous women are more likely to be younger at diagnosis [15].

Through the Delphi process, it became clear that while screening *is* free from 40 years, active invitation does not begin until 50 years and that there is not enough evidence regarding age of incidence in Indigenous women to pinpoint a younger age than 40 years for free screening. It was also acknowledged that the sensitivity of the mammogram decreases the younger the patient, and that in the < 40 years age range there is not much written about screening benefits due to low incidence, comparatively denser breast tissue, and expensive screening tools [16]. The data on mammographic density in Indigenous women suggests that density is a risk factor for breast cancer just as in non-Indigenous women, but overall density tends to be lower. This means that screening with mammography is still a reasonable strategy to use [17]. Our recommendation for screening being free and accessible at a younger age therefore becomes: *Aboriginal and Torres Strait Islander women are actively invited and encouraged to screen from the age of 40 years.*

2. *That women with symptoms are not discouraged from attending breast screening.*

As shown in Table 5, there is a level of complexity around the reasons for discouraging women from screening if they have symptoms. The feedback from the panel on this recommendation was that offering solutions within the recommendation makes more sense than discouragement. However, the compounding effect of discouragement for groups that experience higher barriers to screening is still of concern. It is therefore important that policy clearly includes information regarding the alternatives to screening for women with symptoms.

Newly worded recommendation: *That policy states the pathway women with symptoms of breast cancer should take (instead of only stating they are not appropriate candidates for screening).*

3. *It is not enough to acknowledge the barriers for Aboriginal and Torres Strait Islander women and not address them or provide appropriate solutions.*

There were two policies [18, 19] amongst the complete set of policies which acknowledged and even highlighted the difficulty faced by Indigenous women in screening and treatment of breast cancer. Whilst this acknowledgement is a start, it begs the questions of what can be done about it. These barriers must be addressed in policy. This recommendation was reworded as: *That policy must provide appropriate solutions for the barriers that Aboriginal and Torres Strait Islander women face*.

4. *Policy should not be based on assumption of adherence to guidelines by the consumer.*

Current policy states: “To be effective on a population basis, a high compliance rate of attendance of women in the appropriate age range for screening mammography is necessary” relies on the “compliance” of attendant women. It is not enough for policy to state what works best, apportioning the burden of responsibility to the consumer. Policy should be written for all Australian women and explicitly address access barriers. This recommendation remains the same.

## Discussion

The effectiveness of a policy depends on a number of factors, many of which are government controlled. These include the selection and application of policy instruments in the implementation and operationalisation of policies, the quality of screening equipment and the skill of the readers, decisions about technology, adherence of the women, and developing different approaches for women at very high risk (for example, genetic mutations increasing susceptibility). Our use of the term “high risk” has to carefully distinguish higher incidence (which is not the case with Indigenous women) from higher risk of advanced stage at diagnosis and hence higher mortality.

Policies are how governing bodies set expectations for practice. As previously mentioned, there is a wide body of evidence regarding the poor outcomes for Indigenous women with breast cancer. While there are various guidelines, standards or strategies to improve these outcomes, the evidence is not currently or consistently accounted for in policy. In short, we know what the issues are, we have potential solutions, but we are not implementing these solutions at the policy level.

Throughout the process of undertaking this review, it became clear that Indigenous Australians must be a part of the inception of, consultation for, and writing up of policy, in order for it to better meet the needs of Indigenous women. It is not sufficient to acknowledge poorer outcomes.

### Review of the policy

It appears that policy written regarding breast cancer in Australia is predominantly written for low-risk women and focuses solely on screening and diagnosis (and does not include treatment and follow up care). This comes at a cost for higher risk women whose needs are not adequately met by the policy. Whilst we acknowledge that there are high risk pathways to MRI screening in major public hospitals, these are likely to be difficult to reach for Indigenous patients. There is much anecdotal mention of a lack of resources to tailor policy to the needs of higher risk women; however, this is no excuse for what continues to be poorer outcomes for these women. This review looks beyond whether the policy is a good policy and is concerned with whether the policy meets the needs of those who suffer disadvantage in the larger health context. It builds upon the theory that current policy in Australia does *not* meet the needs of Indigenous women and makes recommendations for how this could be improved.

### Final list of recommendations

1. That Aboriginal and Torres Strait Islander women are consulted in the formation of policy.
2. Aboriginal and Torres Strait Islander women are actively invited and encouraged to screen from the age of 40 years.
3. That policy states the pathway women with symptoms of breast cancer should take (instead of only stating they are not appropriate candidates for screening).
4. That policy must provide appropriate solutions for the barriers that Aboriginal and Torres Strait Islander women face.
5. Policy should not be based on assumption of adherence to guidelines by the consumer.

Our study recognised that there are various guidelines/strategies/action plans produced in an effort to overcome the poorer outcomes for Indigenous people, for example the Optimal Care Pathway for Aboriginal and Torres Strait Islander people with cancer, and that many of these guidelines overlap with the recommendations arising from this review. The issue remains that even with these guidelines, the outcomes are not improving. So while the guidelines and our recommendations align in purpose, they need to be implemented at the policy level.

## Conclusion

Since breast cancer is the most common cancer experienced by Indigenous women in Australia, is diagnosed at an earlier age, and is more likely to be experienced with co-morbidities, it is not surprising that the outcomes are poorer. The evidence indicates that the present approach to breast cancer in the Indigenous population is not making a positive difference. The current breast cancer policy in Australia is written for the majority of Australian women and, while the Indigenous population sits at 3.8% [20], the poorer outcomes for Indigenous women are acknowledged as opposed to addressed at the policy level.

The mortality risk of breast cancer in Indigenous women is higher compared with non-Indigenous women, therefore novel policy strategies are required. This study provides recommendations learned in a review of breast cancer policy in Australia. To address the burden of breast cancer among Indigenous women, this study advocates for the update of breast cancer policy in Australia and for policy makers to work alongside Indigenous communities to ensure that outcomes improve.

## Data Availability

All data generated or analysed during this study are included in this published article [and its supplementary information files].
